# Network inference via adaptive optimal design

**DOI:** 10.1186/1756-0500-5-518

**Published:** 2012-09-24

**Authors:** Johannes D Stigter, Jaap Molenaar

**Affiliations:** 1 Biometris, Wageningen University, Droevendaalsesteeg 1, 6708 PB, Wageningen, The Netherlands

## Abstract

**Background:**

Current research in network reverse engineering for genetic or metabolic networks very often does not include a proper experimental and/or input design. In this paper we address this issue in more detail and suggest a method that includes an iterative design of experiments based, on the most recent data that become available. The presented approach allows a reliable reconstruction of the network and addresses an important issue, i.e., the analysis and the propagation of uncertainties as they exist in both the data and in our own knowledge. These two types of uncertainties have their immediate ramifications for the uncertainties in the parameter estimates and, hence, are taken into account from the very beginning of our experimental design.

**Findings:**

The method is demonstrated for two small networks that include a genetic network for mRNA synthesis and degradation and an oscillatory network describing a molecular network underlying adenosine 3’-5’ cyclic monophosphate (cAMP) as observed in populations of Dyctyostelium cells. In both cases a substantial reduction in parameter uncertainty was observed. Extension to larger scale networks is possible but needs a more rigorous parameter estimation algorithm that includes sparsity as a constraint in the optimization procedure.

**Conclusion:**

We conclude that a careful experiment design very often (but not always) pays off in terms of reliability in the inferred network topology. For large scale networks a better parameter estimation algorithm is required that includes sparsity as an additional constraint. These algorithms are available in the literature and can also be used in an adaptive optimal design setting as demonstrated in this paper.

## Findings

### Background

Recent research in Systems Biology shows that the concept of ‘network’ turns out to be highly relevant at all levels of biological complexity [1,2]. In a modeling approach based on networks, the components of the system under consideration are represented as nodes in a graph and the interactions as edges that connect pairs of nodes. The functioning of a network is determined by its dynamics, i.e., the evolution in time of the nodes. The nodes usually represent the concentrations of some species and their time development is regulated via interactions with other species. At the cellular level, regulatory and metabolic networks are out spoken examples, but also signaling pathways make use of the network concept. Typical examples at the highest complexity levels are predator-prey models and ecological food webs which are in use since long. It is remarkable that not only the network concept forms a unifying element across all biological aggregation levels, but also the mathematics needed to describe the dynamics of the network nodes show great similarities [3]. The central role of network modeling in biology implies that network inference is one of the major challenges of modern biology. The ultimate aim of network inference is to deduce the structure of the network as accurately as possible from data obtained in the experimental practice. To obtain the necessary experimental information, one usually perturbs the network in some way, hoping that the induced response is useful to be explored in some network inference approach. However, if this is not done in a structured way, the harvest might be disappointing. In this paper, we consider the question how we could effectively and fast learn as much as possible of the network structure by designing the experimental setup in an optimal way. It should be realized that the question we put here in a network inference context, is in its generality not new, since similar research is at the core of the mathematical subdiscipline called‘ systems theory’, e.g., [4], and more specifically, in the subdiscipline‘ system identification’ [5]. The fascinating aspect is that insights obtained in the engineering practice may also be of great relevance in a life sciences setting. In an engineering context this kind of research is usually referred to as ‘reverse engineering’. In this paper we also aim at connecting the still too much separated realms of scientists in biology and engineering.

Before getting into details, it is important to remark that network inference may aim at different levels of accuracy [6,7]. The lowest level approach leads to a relatively rough impression of the network structure. Given the nodes, one uses the data to find out whether a coupling exists between any pair of nodes. In mathematical terms, the network is represented as a graph with undirected edges and the inter action is not made explicit, e.g., in terms of an equation. An undirected edge indicates that the dynamics of the two connected nodes are correlated due to some interaction, but the centeracter of this interaction is not pointed out in detail. For example, the first node could affect the second one, but the interaction could also be the other way around. This case is especially relevant if the data contain steady state levels of the nodes obtained after perturbing the system a considerable number of times, e.g., via a knocking out procedure in gene networks. For this case a number of statistical inference methods have been developed that yield undirected graphs as outcome [8-11]. The next level of accuracy is to strive for a directed graph, in which the edges are arrows. The direction of an arrow then indicates a causal relationship [12]. The most sophisticated level is to deduce more detailed information on the centeracter of the interactions. One then tries to answer questions such as: Is it a promoting or an inhibiting interaction (in regulatory networks), is it a linearly increasing, saturating, or decaying interaction (in metabolic networks), etcetera [13,14]. In this paper we aim at the third level of accuracy by including the estimation of the strengths of interactions. A common procedure to generate data is to perturb the system under consideration in a more or less random way. The change in the observed data is then analyzed to infer information about its structure. In practice this may lead to very poor results, since it is not assured that the chosen perturbation is really useful for inference purposes. In this paper we propose to follow a much more advanced approach, in which the perturbations are designed such that they contain the optimal amount of information, given the available data. This approach also starts with a more or less randomly chosen perturbation, but we show that the information from such a first step can be effectively used to design a second perturbation that yields enhanced insight in the network structure. So, we maximize in advance the information content of the second perturbation, given the insight deduced from the first perturbation. This maximization is based on improving the condition number of the so-called Fischer Information Matrix of the system. If required, this procedure could be repeated to refine the inference results further.

Our research focuses on networks whose dynamics are described in terms of ordinary differential equations (ODE). We assume that it is possible to observe the network while it develops in time, so that the data consists of time series of some or all of the network components. The general ideas of the present approach are widely applicable and not at all restricted to a special type of network or level of complexity. For illustrational purposes we will make use of a gene is taken from [15]. This system has been used earlier in several studies to explain and illustrate the principles of other inference methods in signaling and gene networks [16-18] and turns out to be very useful for this purpose. Next, we apply our method to the Laub-Loomis model [19], which describes oscillations in excitable cells of Dictyostelium, and show that our new procedure is very effective in reconstructing the underlying network, just by slightly perturbing system and observing its recovery to its natural oscillatory behavior.

The paper is organized as follows. In the next section we will first introduce two motivating examples in which reconstruction of a directed graph representing the interaction nodes of the network is the primary goal. Then we will further elaborate on the details of adaptive optimal input design [20] and explain the method. Finally, the methodology will be applied to the two motivating examples explained earlier and some results will be presented and discussed.

### Methods

#### Two motivating examples

In this section we introduce two biological systems that will be used later on to illustrate how our adaptive optimal design approach works in practice. For both models centeracteristic parameter values are taken from the literature. These values are used to generate artificial data and to mimic real practice these data are spoiled by adding noise. The present aim is to show how the parameters can be efficiently estimated from the data in an adaptive manner.

##### mRNA synthesis and degradation

Let us consider the well-known Kholodenko case as a motivating example [15]. It consists of a small four gene network and the gene activity is reflected by the synthesis and degradation of mRNA as follows:

(1) $${\dot{x}}_{1}\left(t\right)=\nu_{\text{syn},1}-\nu_{\text{deg},1}$$

(2) $${\dot{x}}_{2}\left(t\right)=\nu_{\text{syn},2}-\nu_{\text{deg},2}$$

(3) $${\dot{x}}_{3}\left(t\right)=\nu_{\text{syn},3}-\nu_{\text{deg},3}$$

(4) $${\dot{x}}_{4}\left(t\right)=\nu_{\text{syn},4}-\nu_{\text{deg},4}$$

where $\dot{x}\left(t\right)$ stands for differentiation of x(t) with respect to time [h], and the synthesis and degradation rates are given by the following non-linear relationships:

(5) $$\nu_{\text{syn},1}=V_{1\text{s}}\frac{1+A_{14}{\left(x_{4}/K_{14a}\right)}^{n_{14}}}{\left(1+{\left(x_{4}/k_{14a}\right)}^{n_{14}}\right)\left(1+{\left(x_{2}/k_{12i}\right)}^{n_{12}}\;\right)}$$

(6) $$\nu_{\text{syn},2}=V_{2\text{s}}\frac{1+A_{24}{\left(x_{4}/K_{24a}\right)}^{n_{24}}}{\left(1+{\left(x_{4}/K_{24a}\right)}^{n_{24}}\right)}$$

(7) $$\nu_{\text{syn},3}=V_{3\text{s}}\frac{1+A_{32}{\left(x_{2}/K_{32a}\right)}^{n_{32}}}{\left(1+{\left(x_{2}/K_{32a}\right)}^{n_{32}}\right)\left(1+{\left(x_{1}/K_{31i}\right)}^{n_{31}}\;\right)}$$

(8) $$\nu_{\text{syn},4}=V_{4\text{s}}\frac{1+A_{43}{\left(x_{3}/K_{43a}\right)}^{n_{43}}}{\left(1+{\left(x_{3}/K_{43a}\right)}^{n_{43}}\right)}$$

(9) $$\nu_{\text{deg},1}=V_{1\text{d}}\frac{{\text{x}}_{1}\left(t\right)}{{\text{x}}_{1}\left(t\right)+K_{1\text{d}}}$$

(10) $$\nu_{\text{deg},2}=V_{2\text{d}}\frac{{\text{x}}_{2}\left(t\right)}{{\text{x}}_{2}\left(t\right)+K_{2\text{d}}}$$

(11) $$\nu_{\text{deg},3}=V_{3\text{d}}\frac{{\text{x}}_{3}\left(t\right)}{{\text{x}}_{3}\left(t\right)+K_{3\text{d}}}$$

(12) $$\nu_{\text{deg},4}=V_{4d}\frac{{\text{x}}_{4}\left(t\right)}{x_{4}\left(t\right)+K_{4d}}$$

The values of the parameters in these expressions are presented in Table 1.

**Table 1:** Parameter values mRNA synthesis and degradation

						
*V*_1*s*_ = 5	*A*_14_ = 4	*K*_14*a*_ = 1.6	*K*_12*i*_ = 0.5	*n*_12_ = 1	*V*_1*d*_ = 200	*K*_1*d*_ = 30
*V*_2*s*_ = 3.5	*A*_24_ = 4	*K*_24*a*_ = 1.6	*n*_32_ = 2	*n*_14_ = 2	*V*_2*d*_ = 500	*K*_2*d*_ = 60
*V*_3*s*_ = 3	*A*_32_ = 5	*K*_32*a*_ = 1.5	*K*_31*i*_ = 0.7	*n*_31_ = 1	*V*_3*d*_ = 150	*K*_3*d*_ = 10
*V*_4*s*_ = 4	*A*_43_ = 2	*K*_43*a*_ = 0.15	*K*_12*i*_ = 0.5	*n*_43_ = 2	*V*_4*d*_ = 500	*K*_4*d*_ = 50

For simplicity we start by assuming that each time an experiment starts them RNA transcription and degradation rates are in equilibrium, i.e. there is a steady state for all four states in the model. This means${\dot{x}}_{1}\left(t\right)={\dot{x}}_{2}\left(t\right)={\dot{x}}_{3}\left(t\right)={\dot{x}}_{4}\left(t\right)=0$. Furthermore, we assume, just as in the original paper, that we can influence the maximum transcription rates *{V*_*is*_*, i*=1*,…,*4*}* thereby silencing the synthesis of mRNA at will. In other words, the maximum transcription rates represent an input signal that we can manipulate (or perturb) in such a way that information regarding the structure of the network is revealed through the stimulus-response data. An interesting (and challenging) aspect is that, in practice, it is *not* known what the exact values of the input signals are. Put differently, the (constant) input stimuli (or excitation signals) are in this case a natural part of the identification problem and these values are included in the parameter estimation problem. The ODE system in equations (1)–(12) can be written in the general form

(13) $$\dot{x}=\text{f}\left(\text{x},\theta ,\text{u}\right)$$

Here, **x** is the state vector **x** = (*x*_1_*,x*_2_*,x*_3_*,x*_4_)^T^ , **u** is the vector containing the inputs, so **u** = (*V*_1*s*_*,V*_2*s*_*,V*_3*s*_*,V*_4*s*_)^T^ ,*θ* is the vector containing the remaining parameters in Table 1, and **f** is the vector valued function defined in equations (5)–(12).

If we linearise the above equations and evaluate the resulting equations at the steady state **x**_*ss*_ a *linear* state space model is obtained that reads as

(14) $$\frac{\text{d}\left(\delta \text{x}\right)}{dt}=a\;\delta \text{x}\left(t\right)+\text{B}\;\delta \text{u}$$

where *δ*x(*t*) and *δ***u** are now the deviations from the steady state **x**_*ss*_ and the corresponding (steady) input **u**_*ss*_ that maintains this steady state. The matrix **A** in equation (14) contains the so-called interaction coefficients of the network and these correspond to a *directed* graph where there is an interaction *from* node *j to* node *i* if the corresponding matrix element *a*_*ij*_*≠* 0. We note that, of course, this linearisation is a *local* property that depends on our choice of the linearisation point in state space where we derive the linear system (14). However, it is important to realize that, if nodes *i* and *j* are not connected, the matrix **A** has zeros at positions *ij* and *ji*, regardless of the linearisation point that is used! The question of optimal experimental design now comes down to choosing the entries of the vector *δ***u** in such a way that matrix pair [A*,*B] can be estimated in an optimal manner.

##### Laub-Loomis model–the no-input case

A well-known example of an oscillating network in systems biology is Laub-Loomis’ model of the molecular network underlying adenosine3’-5’-cyclic monophosphate (cAMP) as observed in populations of Dyctyostelium cells [19]. The model incorporates changes in the activities of cAMP and consists of a set of seven ordinary differential equations (one for each state) that reads

(15) $${\dot{x}}_{1}\left(t\right)=k_{1}x_{7}-k_{2}\;x_{1}x_{2}$$

(16) $${\dot{x}}_{2}\left(t\right)=k_{3}x_{5}-k_{4}x_{2}$$

(17) $${\dot{x}}_{3}\left(t\right)=k_{5}x_{7}-k_{6}\;x_{2}\;x_{3}$$

(18) $${\dot{x}}_{4}\left(t\right)=k_{7}-k_{8}\;x_{3}\;x_{4}$$

(19) $${\dot{x}}_{5}\left(t\right)=k_{9}-k_{10}x_{4}x_{5}$$

(20) $${\dot{x}}_{6}\left(t\right)=k_{11}x_{1}-k_{12}x_{6}$$

(21) $${\dot{x}}_{7}\left(t\right)=k_{13}{x_{6}}_{-}k_{14}x_{7}$$

where the parameter values for the constants *{k*_*i*_*,i*=1*,…,*14*}* are given in Table 2. For these parameter values the system shows oscillating behavior.

**Table 2:** Parameter values Laub–Loomis

						
*k*_1_ = 2.0	*k*_2_ = 0.9	*k*_3_ = 2.5	*k*_4_ = 1.5	*k*_5_ = 0.6	*k*_6_ = 0.8	*k*_7_ = 1.0
*k*_8_ = 1.3	*k*_9_ = 0.3	*k*_10_ = 0.8	*k*_11_ = 0.7	*k*_12_ = 4.9	*k*_13_ = 23	*k*_14_ = 4.5

In the first motivating example we used the maximum synthesis rates as system inputs. Here, we assume that this oscillatory system can be perturbed by kicking it out its steady limit cycle. Since this cycle is asymptotically stable, the system will converge back to it. It is just the way in which this return happens, that provides us with the additional (and necessary) information to estimate the network structure. The linearised version of the Laub-Loomis model reads

(22) $$\frac{\text{d}\left(\delta \text{x}\right)}{dt}=a\;\delta \text{x}\left(t\right)$$

If we allow a Dirac pulse, we simulate a sudden change in the state vector values so that the system is ‘kicked’ from its steady oscillation and needs to recover from this ‘shock’, returning (after some time) to its initial oscillatory behavior. Put differently, in mathematical terms we allow ‘inputs’ to be of the form **b***δ* (*t*), where **b** is a vector with dimensions equal to the state vector x(*t*), so that

(23) $$\frac{\text{d}\left(\delta \text{x}\right)}{dt}=a\;\delta \text{x}\left(t\right)+\text{b}\;\delta \left(t\right)$$

If we assume the perturbation *δ****u*** to be a Dirac delta function (or better, a *δ*-distribution), the question of optimal experimental design now becomes one of finding the values of the entries in the vector **b** that yield an optimal estimate **A**, i.e., an estimate Â with lowest possible uncertainty bounds.

#### Parameter estimation– towards adaptive experimental design

**Figure 1:**
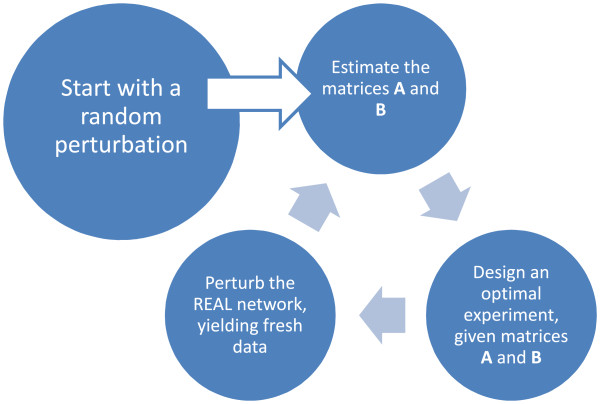
**The adaptive input design loop.** Figure 2 represents the adaptive input design methodology as presented in this paper in a flow-centert.

For a given choice of our input perturbation, e.g., *δ****u*** in our first motivating example that contains four silencing-percentages for each of them RNA synthesis processes, we now perform a simple step response experiment for each of the four inputs *{u*_*i*_ (*t*)*, i*=1,*…*,4*}* and this yields an observation record of mRNA concentrations. In Figure 1 we see an example of such data that includes 20% error in the observed mRNA concentrations after a random relative perturbation (between-50% and 50%) of all input parameters *{V*_*is*_*, i* = 1*,…,*4*}* in the original (non-linear) model.

Since there are four parameters*{V*_*is*_*, i* = 1*,…,*4*}*, there are four ‘inputs’ at our disposal that we can use as perturbation candidates. In the following we assume that four separate perturbations of *all* inputs are generated with random values between _*−*_50% and 50% of the steady state input values contained in the vector **x**_*ss*_ (that corresponds to the initial steady state **u**_*ss*_).

Schmidt *et al.*[16], inspired by the methods and results of [15], applied linear regression to data obtained by virtual experiments using the original system model as a simulation tool. Based on calculated deviations from the steady state **x**_*ss*_ a linear regression formula was obtained with the coefficients of matrices **A** and **B** as unknowns. Schmidt successfully computed the interaction coefficients, including the unknown input perturbations, from step-response data. For clarity it is noted that the step-response data represents the *dynamic* transient behavior from one steady state to another steady state. This is different from the approach presented by Steinke and co-workers [21], where an interesting *statistical* methodology was applied with the same idea of iterative experimental design in mind, but for the *steady states* only, i.e. with no dynamic transient behavior included.

It should further be noted that the regression method applied here only works for relatively small scale problems in which the number of states is not too large. This is mainly because of the limitations in the applied parameter estimation method (see below for details) which easily introduces identifiability problems for large scale networks where the number of parameters to be estimated in the Jacobi matrix is simply too large. The disadvantage of ordinary least squares estimation, however, can be remedied for larger scale networks by more advanced parameter estimation algorithms that include *sparsity* of the network as an additional constraint (see e.g. [22] for a convex linear-programming approach.)

A discretized version of the linear model (14) with sampling interval Δ*t*=*t*_*k*+1−_*t*_*k*_*reads*

(24) $$\text{x}\left(k+1\right)=\Phi \text{x}\left(k\right)+\Gamma \text{u}\left(k\right)$$

(25) $$=\left(\Phi \;\Gamma \right)\left(\begin{array}{c} x\left(k\right)\\ u\left(k\right) \end{array}\right)$$

with (see e.g. [4])

(26) $$\Phi ={\text{e}}^{a\;\Delta \text{t}}$$

(27) $$\Gamma =\int_{t_{k}}^{t_{k+1}}{\text{e}}^{a\left(t_{k+1}-\tau \right)}\text{Bu}\left(\tau \right)d\tau$$

In summary the parameter estimation method proceeds as follows:

Step 1. Collect *N* observations of the state vector **x** in the original non-linear model (13) with a sampling interval Δ*t*. Subtract the steady state **x**_**ss**_ from these observations.

Step 2. Calculate the difference between consecutive time instances of the obtained measurements in step (1) and stack these differences in a (*n* × (N _−_ 1)) matrix **M** with *n* the dimension of the state vector x and *N* the number of data points.

Step 3. Take **M**_**n**_ as the 2^*nd*^ until the last column of **M** and take **M**_**o**_ as the 1^*st*^ until the last but one column of M. Here, the subscripts *n* and *o* stand for *new* and *old* to denote a cause-effect relation between the matrices **M**_**n**_ and **M**_**o**_.

Step 4. Augment the matrix **M**_**o**_ with a (1 _×_ (N _−_ 1)) row of 1’s. This is to estimate the input sequence *{*Δu(*k*) = Γu(*k*)*,k* = 0*,…,N*_−_ 1*}*. Denote the resulting augmented matrix with **M**_**a**_.

Step 5. An estimate of the matrices Φ and Γ is now calculated via linear regression (see [16]) as

(28) $$\left(\hat{\Phi }\;\hat{\Gamma }\right)=M_{n}{M_{a}}^{T}{\left(M_{a}{M_{a}}^{T}\right)}^{-1}$$

Step 6. Translate the discrete time result $\hat{\Phi }\;\hat{\Gamma }$ back to continuous-time (see e.g. the standard textbook [23]), yielding the estimates $\hat{A}$ and $\hat{B}$.

**Figure 2:**
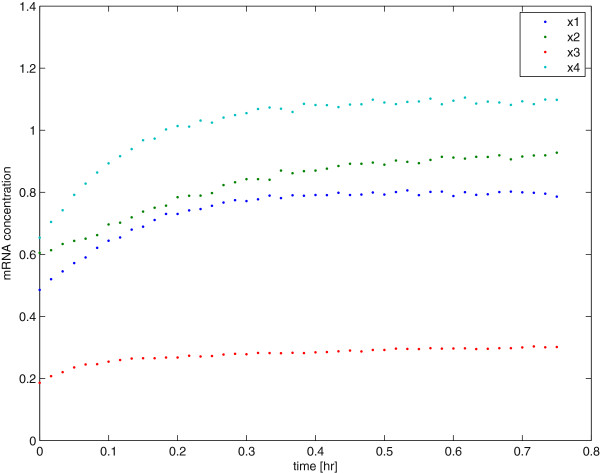
**Time series data of a step response for the Kholodenko network model.** Time series for the variables *x*_1_*,…,x*_4_ in equations (1)–(4) after a random perturbation of the parameter *V*1*s*. We can clearly observe step-response curves in these data.

At this point it should be mentioned that the familiar parameter estimation method in the above does not explicitly include any analysis of the *uncertainty* in the parameter estimates obtained, although this is of course an important measure of the quality of the parameter estimates that have been obtained using the simple regression formulae (28). Our goal in the current paper is to perform *model based experiments* that minimize the uncertainty bounds on the parameter estimates ‘on-the-fly’. To explain best what we mean with this the reader is referred to Figure 2 in which the adaptive optimal input design methodology is depicted as an iterative loop. Starting with an initial random perturbation/experiment one moves on to a first parameter estimate $\hat{A}$, $\hat{B}$ of the Jacobi matrices **A**, **B** following the 6-step procedure out lined in the above. Since at this point an *approximate* model is available, one can progress by designing a new perturbation/experiment in such a way that a norm of the so-called Fisher Information Matrix (FIM) is optimized. The FIM incorporates the parametric output sensitivities $\frac{dy\hat{T}}{d\theta \left(\tau ;\theta \right)}$, where **y** is the output vector which, in the two case studies discussed in this paper equals the state vector **x** since all states are assumed measurable with measurement error ∈(**k**), (*k*=1*,…,N*), that is assumed to be sampled from a Gaussian white noise sequence with co-variance matrix **Q**. We note that the parametric output sensitivity $\frac{dy\hat{T}}{d\theta \left(\tau ;\theta \right)}$ can be calculated via the original state space model (13) (see e.g.[20]) through simple differentiation of the state equations with respect to the parameter vector *θ*. The FIM represents a measure of the information content of the given input-output data with regard to the parameter values of the parameters *θ* in the model. We choose to minimize the so-called modified E-norm of the FIM [24], which is defined as the ratio of the maximum eigenvalue of the FIM by its minimum eigenvalue, i.e.,

(29) $$F\left(t_{f};\hat{\theta }\right)=\int_{0}^{\mathrm{tf}}\frac{dy\hat{T}}{d\theta \left(\tau ;\theta \right)}{\text{Q}}^{-1}\frac{dy\hat{T}}{d\theta \left(\tau ;\theta \right)}d\tau$$

(30) $$||F\left(t_{f};\hat{\theta }\right)|{|}_{\mathrm{Emod}}=\frac{\max\left\{\lambda_{F}\right\}}{\min\left\{\lambda_{F}\right\}}$$

where *t*_*f*_ marks the duration (final time) of our experiment and where we have denoted with $\hat{\theta }$ the fact that the FIM can only be calculated based upon the best knowledge we have available of the parameter values and *not* based upon the true values θ*. In practice this optimization can be performed crudely by evaluating a large number, say *N* = 1000, of random perturbations (through simulation of the approximate model (13) with the pair $\hat{A},\hat{B}$) and evaluate the modified E-criterium for each of these perturbations. Once the *N* performances have been calculated we choose the perturbation that is associated with the minimum of the *N* modified E-criteria. For clarity we note that optimizing the FIM with regard to some norm can be seen as away to better condition the matrix inversion in equation (28). For information rich experiments the matrix **M**_**a**_**M**_**a**_^***T***^ can be shown to be better conditioned and, hence, the inversion is less prone to numerical errors that can easily result in case of a badly condition matrix.

Having thus progressed to the bottom-right circle in Figure 2, we continue to apply the ‘optimal’ perturbation to the *real* system, yielding an additional set of observations that can now be used for a second parameter estimation exercise. We have now turned full circle and can start again with an improved estimate of the system **A**, **B**, i.e. ${\hat{A}}^{1},{\hat{B}}^{1}$. Several iterations of the loop in Figure 2 will now yield parameter estimates that have been inferred from an increasingly rich set of observations. In other words, we have maximized the information content of the model parameters to the best of our (current) knowledge of the network as to converge rapidly to the true parameter values contained in the matrix-pair **A**, **B**. Through minimization of the uncertainty bounds on the parameter estimates via optimal input design a better conditioned parameter estimation problem is gained and this is highly preferred above the ad-hoc approach where random perturbations are used as an experimental ‘design’. Although the above methodology (and more specifically the parameter estimates that are obtained using simple linear regression as in [16]) can only be used for relatively small networks, the adaptive input design approach can easily be extended to larger scale problems if other parameter estimation algorithms are utilized that can better handle larger scale networks (see e.g. [22] for a linear-programming approach to the parameter estimation problem for large networks). The message we would like to convey in the current paper is not about a parameter estimation algorithm, but about utilizing such an algorithm in the best possible way as to obtain reliable parameter estimates.

### Results

#### Kholodenko case study

On the basis of a random perturbation (with maximum perturbations of 50% for all four maximum transcription rates in the original non-linear model) we obtained artificial time series for all four components of **x**, i.e. the four mRNA concentrations, with a time-interval Δ*T* = 1 minute and for a length of 0*.*5hr, so that *N* = 30. We added 5% noise on these readouts. Furthermore, we assumed the matrix **B** to be diagonal. This implies that the perturbations *δu* influence the corresponding mRNA state *directly*. The first data sets were generated by performing 5 *random* perturbation experiments in a row and estimating the matrix **A** from these 5 experiments, yielding an estimate for **Â** that is summarized in equation (33). The numerical values for **Â** (deduced from 5 random experiments) together with their true values are:

(31) $$A=\left(\begin{array}{c} -6.45\\ 0.00\\ -2.31\\ 0.00 \end{array}\begin{array}{c} -2.92\\ -8.17\\ 2.80\\ 0.00 \end{array}\begin{array}{c} 0.00\\ 0.00\\ -14.46\\ 10.22 \end{array}\begin{array}{c} 2.54\\ 3.93\\ 0.00\\ -9.74 \end{array}\right)$$

(32) $$\hat{A}=\left(\begin{array}{c} -7.15\\ -0.78\\ -1.51\\ -1.74 \end{array}\begin{array}{c} -2.53\\ -8.19\;\\ 1.63\\ 1.65 \end{array}\begin{array}{c} -1.69\\ 0.039\\ -8.59\\ 0.37 \end{array}\;\begin{array}{c} 3.20\\ 5.83\\ 0.23\\ -8.56 \end{array}\right)$$

The idea is now to try to improve the parameter estimates by performing an input design based on the model (13) with the initial estimates $\hat{A}$ and $\hat{B}$that we obtained using the first data set–meaning that we used the *first* of the 5 random experiments to find a first estimate **Â** for an input design for the second experiment. We then applied this procedure sequentially, i.e. the “optimal” input found from the input design was fed to the original non-linear system model to create a new data set and this data set was again used to estimate **Â**, resulting in a second estimate of the Jacobi matrix, etc. This procedure was repeated until 5 experiments (using OID for the second to fifth experiment) were computed. The final values for the matrix entries in **Â** were found to be:

(33) $$\hat{A}=\left(\begin{array}{c} -6.50\\ 0.68\\ -3.05\\ 0.91 \end{array}\begin{array}{c} -3.14\\ -7.66\\ 3.91\\ -1.45 \end{array}\begin{array}{c} 1.36\\ 3.20\\ -15.18\\ 10.25 \end{array}\begin{array}{c} 2.43\\ 3.15\\ 0.26\\ -9.54 \end{array}\right)$$

**Figure 3:**
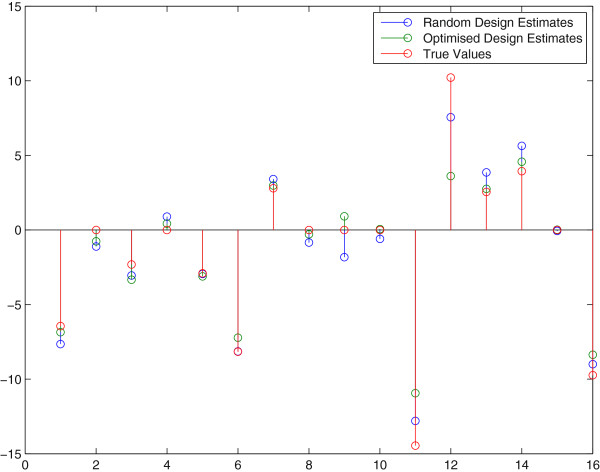
**Parameter estimates drawn from a random perturbation experiment and an optimized perturbation experiment.** Parameter estimates (and true values) for a random and an optimized experiment that was designed on the basis of the first estimate of **A**. The sixteen parameters are all entries in the Jacobian matrix **A**. We observe a substantial improvement of the parameter estimates once model based design was performed.

**Figure 4:**
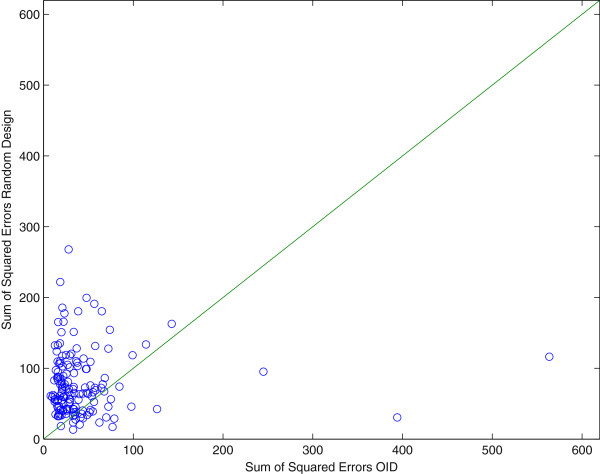
**Comparison plot of Random Design versus Optimal Input Design (OID).** This graph shows the sum-of-squared-errors in the parameters (Jacobi matrix entries) for 150 runs of our algorithm for a series of 5 experiments. Each run is a point in this graph with coordinates (Random, OID) where Random stands for the sum-of-squared-errors for the Random run and OID stands for the sum-of-squared-errors for the OID run that started with the same initial estimate of the matrix **A** that was obtained in the first random experiment.

These results have also been included in Figure 3 which shows a comparison between the final estimate of the two approaches (OID and random). As to better get insight in the success rate of OID we repeated the above procedure 150 times. In Figure 4 a comparison of the two methods is presented in a graph to see the difference in performance. Each point in this graph represents a pair of sum-of-squared-errors (of the parameters in the Jacobi matrix) for an optimal input design (x-coordinate) versus a random design (y-coordinate) series of 5 experiments in a row, repeated for 150 trials. The line *y* = *x* separates the two cases and we found that for 82% of the 150 pairs (OID, Random Design), the OID results were better, i.e. the x-coordinate was *smaller* than the y-coordinate. Apparently, random design can still be better than OID in 18% of the cases and this can be understood since a wrong first estimate **Â** in the first OID experiment means that we design inputs based on erroneous information that points us in the wrong direction for designing a better experiment. These results corroborate with the fact that if we reduce the measurement uncertainties on the sensors, OID becomes even better than Random Design because it has a better starting point in the series of 5 experiments. More specifically, after reducing the measurement error to only 1%, the success rate of OID versus Random was 91%.

#### Laub-Loomis case study

The Laub-Loomis case study presents us with a far more challenging problem in comparison to the Kholondenko case study. This is mainly because in this particular case study 49 entries in the Jacobi matrix have to be estimated with linear regression, which is a large number. Furthermore, the non-linearity of the model introduces additional difficulties. It is therefore required to use relatively small perturbations in the initial condition **x**(0) as to not divert too far from the linearity assumption that underpins the linearized perturbation model. Since small perturbations are difficult to trace back if the noise on the data is too high, we are certainly limited in our possibilities.

**Figure 5:**
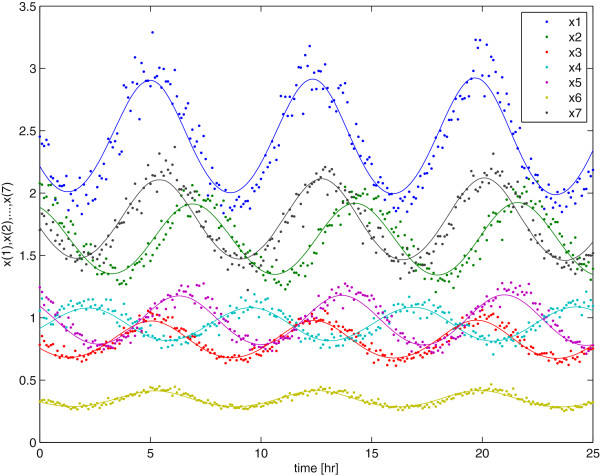
**Response of the Laub-Loom is model for a random initial perturbation.** Oscillatory behavior after a random initial perturbation. The solid lines represent unperturbed behavior while the dotted lines represent oscillations due to a small perturbation in the initial condition.

To perturb the oscillatory Laub-Loomis model we apply a ‘shock’-effect to each of the entries in the state vector **x**(*t*) at time *t* = 0 and observe how the system recovers to its normal oscillatory behavior. An example of data from a random initial perturbation are presented in Figure 5. For the sampling interval of this observation record we assumed Δ*t* to be 0.1 hr. Furthermore, the data set includes 20% artificial noise on the deviated observations, i.e. the observed value x_*obs*_ minus its nominal value x_*nom*_ where the nominal value refers to an unperturbed oscillatory behavior. It can be observed in Figure 5 that a recovery of the initial perturbation to a ‘steady’ oscillatory behavior (solid lines in figure) is present.

**Figure 6:**
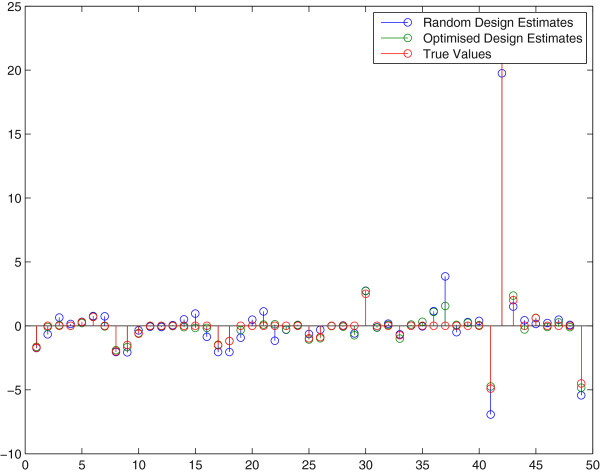
**Parameter estimates drawn from an optimized perturbation experiment.** Parameter estimates (and true values) for a random experiment and an optimized experiment that was designed on the basis of the first estimate of **A**. The parameters are all entries in the Jacobian matrix **A**. We again observe improvement of the parameter estimates once model based design was performed.

We found in the initial run that the estimated parameter values are still far off from the true parameter values. In order to find better estimates these initial estimates are now used for the next experimental design. Hereto the model (22) was evaluated for 250 random perturbations and for each of those 250 (virtual) experiments a modified E-criterion for the FIM was calculated. Taking the minimal value of these E-criteria lead to an improved perturbation that was subsequently applied to the real system (15)–(21). The second data-set was then used in a second parameter estimation exercise and OID procedure was repeated another time so that 3 experiments in total were used. In Figure 6 we see the final estimation results after 1 random and 2 OID experiments. These are compared with a complete random experiment series of 3 experiments in total. Again, we see an improved parameter estimation result–almost all parameter estimates are closer to their true values in comparison with the random parameter estimation result.

**Figure 7:**
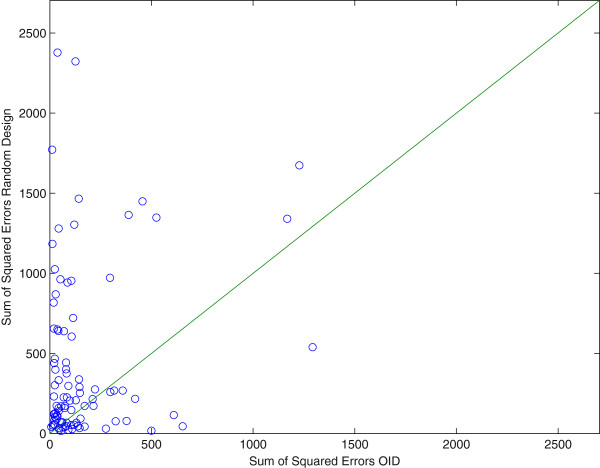
**Comparison plot of Random Design versus Optimal Input Design (OID).** This graph shows the sum-of-squared-errors in the parameters (Jacobi matrix entries) for 100 runs of our algorithm for a series of 3 experiments. Each run is a point in this graph with coordinates (Random, OID) where Random stands for the sum-of-squared-errors for the Random run and OID stands for the sum-of-squared-errors for the OID run that started with the same initial estimate of the matrix **A** that was obtained in the first random experiment.

The above procedure of simulating three experiments and apply random design versus OID was then repeated 100 times as to better compare the OID approach to a completely random approach. In Figure 7 we see a comparison graph for pairs (OID, Random) together with the line *y* = *x* that represents the line of equal performance. From the 100 trials (of three experiments each) we found that 74% of the OID results were better than a completely random design. In addition the average sum-of-squared-errors of the estimates of the entries in the Jacobi matrix were 214 for OID and 745 for the random experimental design, meaning that, on average, in this particular case OID performs three times better in terms of average uncertainty on the parameter estimates. This is certainly significant, even more after realizing that the Jacobi matrix is time-varying and 49 estimates need to be recovered from the noised at a provided. We also tried other noise-levels and sampling frequencies and found that when the noise on the data increases to 50% or more, there is no distinction between the random design and OID visible. Clearly, the signal to noise ratio is so low in these cases that the parameter estimation algorithm cannot recover more information from the data on the basis of OID. But in such cases where the noise levels are just too high, clearly, there is not much to be gained from any approach since the information content of the data set is so low.

### Discussion and conclusions

Adaptive optimal experimental design is a natural approach for the recovery of the connections in an interaction network based on a limited number of experiments. Clearly, the methodology as presented in this paper yields promising results as was demonstrated in two case studies. The idea we have pursued is that adaptive or sequential input design closes the model identification loop of experimentation and subsequent calibration of the model, intelligently taking in to account the most recent knowledge that is available. More specifically, this means that the most recent parameter estimate of the Jacobi matrix pair [A*,* B] are used for subsequent analysis of the uncertainty propagation in the network. An essential feature proposed in our methodology (and also demonstrated in two case studies) is that model based experiments are now prominently included in the loop and here with the best knowledge that is available, i.e. the most recent estimate [$\hat{A},\hat{B}$] of the parameter vector *θ*. This yields a more carefully designed parameter estimation problem that allows a more reliable network reconstruction.

One could argue that the linearized system introduces limitations since, of course, the matrices **A** and **B** depend on the linearization point chosen. Especially if the Jacobi matrix structure changes significantly (in terms of zeros and non-zeros) this can introduce a problem. A remedy would then be to repeat the algorithm for different values of the state vector **x**. However, we emphasize that if there is no connection between node *i* and *j* in the network then regardless of the linearization point there will always be a zero in the *ij*^*th*^ entry. If, therefore, a zero is present in more than one linearization point, the chances are increasingly high that there is indeed no connection between node *i* and *j* in the network structure. Our approach contributes to the field of systems biology where the recovery of networks based on stimulus-response data is a central topic that has already caught a lot of attention [6,7]. In systems engineering, model based experimentation has matured substantially but here the design is usually developed for recovery of a set of model parameters in the *original* non-linear model structure and not for network inference, see e.g. [20] where the input design is formulated in a recursive manner, meaning that the data and subsequent planning of a new experiment are processed/analysed on-line. Of course, the results presented in the current paper do not include large scale networks with several hundreds of differential equations. But, then, this is not our main message. Rather, we have shown that OID based on *dynamic* time series of simple perturbation experiments leads to a better overall performance of the parameter estimation algorithm. In addition, the idea of adaptive input design may very well be combined with parameter estimation algorithms that are especially geared for such large scale problems and we think this will almost certainly improve the efficiency of subsequent experimentation and calibration as a means to unravel the underlying structure in a network model of a biological system.

## Competing interests

The authors declare that they have no competing interests.

## Author’s contributions

JD Stigter performed the calculations in the two case studies and wrote the main text. J M wrote most of the introduction and edited the remaining text. Both authors developed the ideas and frame work underlying this paper. Both authors read and approved the final manuscript.
